# Editorial Notes

**Published:** 1874-09

**Authors:** 


### Editorial.

---

We are glad to learn from a correspondent that the physicians of Hernando, Hillsboro, Manatee and Polk counties, in the State of Florida, have organized themselves into the "South Florida Medical Society," auxiliary to the State Medical Association. The officers elected are: President, Dr. D. Branch, of Tampa; Corresponding Secretary, Dr. John P. Wall, of Tampa; Recording Secretary and Treasurer, Dr. A. S. Johnson, of Bartow. We bid them God-speed in the good work, and trust we may be able to present to our readers in the future some valuable papers from their transactions.

---

We cheerfully yield our editorial space this month that the unusual number of original communications on hand may find admittance.

---

The numbers of this Journal for August, September and October, 1861, are wanted to fill the files of the Library of the Surgeon General's office at Washington. A liberal price will be paid for them.

The Library needs also the annual announcements of the Atlanta Medical College for the years 1855, 1856, 1857, 1860, 1866, 1869, 1870 and 1871. We trust our subscribers will look over their old files, and endeavor to supply these deficiencies, if practicable, and thus aid in building up a great national medical library.

---

We have received the first number of another candidate for the patronage of Southern medical men, namely: *The Louisville Medical Reporter; a Weekly Journal.* Editor, J. L. Cook, M.D.; Associate Editor, James M. Holloway, M.D.; published at Henderson, Ky., at $3.50 per year.

The *Reporter* is neatly gotten up in tinted cover, and promises to be a welcome weekly visitor to its subscribers. We heartily wish the editor and publisher abundant success in his new enterprise.

We have received the prospectus of the Archives of Dermatology; a quarterly journal of Skin and Venereal diseases. L. Duncan Bulkley, A.M., M.D., editor. It is to be published in New York, by G. P. Putnam's Sons, 4th Avenue and 23d Street, at three dollars per annum.

The high reputation, both of the editors and the publishers, gives assurance that the Archives will be the standard journal of this country in its special department.

---

Murderous Attack by Lunatics.—A terrible event is announced as having occurred at the Lunatic Asylum of St. Andrews, near St. Petersburg, Russia. While the keepers were at dinner, the patients burst into a room where arms were stored, and with them attacked the warders, five of whom were killed and two seriously wounded. Six of the most violent of the assailants were placed in strait-waistcoats and confined in separate cells.

Pills of Sulphate of Quinia.—Mr. A. Schreiber, of Tell City, Indiana, informs us that a smaller quinia pill can be made by using a mixture of equal parts of glycerine and muriatic acid, than with the glycerine alone. Mr. S. has used such a mixture for this purpose for several years.—*American Journal of Pharmacy.*

